# LncRNA MALAT1 Suppression Protects Endothelium against oxLDL-Induced Inflammation via Inhibiting Expression of MiR-181b Target Gene TOX

**DOI:** 10.1155/2019/8245810

**Published:** 2019-12-14

**Authors:** Liuqing Wang, Yinliang Qi, Yi Wang, Haitao Tang, Zhenzhen Li, Yuan Wang, Songtao Tang, Huaqing Zhu

**Affiliations:** ^1^Department of Clinical Laboratory, The Third Clinical School of Heifei of Anhui Medical University, Hefei 230051, China; ^2^General Department of Hyperbaric Oxygen, Hefei Hospital Affiliated to Anhui Medical University, Hefei 230011, China; ^3^Laboratory of Molecular Biology and Department of Biochemistry, Anhui Medical University, Hefei 230032, China; ^4^Department of Endocrinology, The First Affiliated Hospital of Anhui Medical University, Hefei 230022, China

## Abstract

Rare studies have been conducted to investigate the exact interactions between lung adenocarcinoma transcript 1 (MALAT1), thymocyte selection-associated high mobility group box (TOX), and miRNAs in the pathogenesis of atherosclerosis (AS). We aim to investigate the crosstalk between MALAT1 and TOX and evaluate whether the regulatory mechanism was associated with the miRNA network. AS tissues were collected to determine the level of MALAT1 expression in AS patients, together with determination of miR-181b expression. Cultured endothelial cells were utilized to analyze the expressions of MALAT1, miR-181b, and TOX in the presence of oxLDL. Luciferase activity assay was conducted to evaluate the potential target sites of miR-181b on MALAT1 and TOX. In this study, we demonstrated that MALAT1 was upregulated in patients with AS. MALAT1 silencing significantly downregulated the expression of the miR-181b target gene TOX via reversing the effect of miR181b. Importantly, positive modulation of miR181b and inhibition of MALAT1 and TOX significantly attenuated oxLDL-induced endothelial inflammation and oxidative stress. Moreover, the MAPK signal pathways in endothelial cells were also inhibited through regulation of above endogenous RNAs. In summary, MALAT1 suppression protects the endothelium from oxLDL-induced inflammation and oxidative stress in endothelial cells by upregulation of miR-181b and downregulation of TOX.

## 1. Introduction

Atherosclerosis (AS), induced by plaque formation inside the arteries, is a lethal condition responsible for heart attack and stroke [1, 2]. Currently, AS has been closely related to the pathogenesis of cardiovascular diseases (CVDs), serving as the most common cause for death [3, 4]. Oxidized low-density lipoprotein (oxLDL) has been widely demonstrated to be involved in the development of AS by causing an oxidative chain reaction and inducing endothelial dysfunction. However, its exact mechanism is not well defined.

MicroRNAs (miRNAs), a class of small noncoding single-stranded RNA, have been reported to negatively regulate the gene expression by degradation or posttranscriptional regulation of target sequences. Several miRNAs have been considered to participate in the pathogenesis of AS. For instance, miR-27b is a cholesterol-responsive hepatic miRNA that represses a large number of targets involving in lipid metabolism and lipoprotein remodeling that play important roles in AS [5]. MiR-146a is an important cytokine-responsive miRNA conferring atheroprotective properties in vessel walls [6]. In addition, miR-146a showed elevation in atherosclerotic plaques of human and mouse [7]. To date, increasing evidence shows that miR-181b plays a critical role in mice and human subjects by serving as an inhibitor of endothelial inflammatory responses through targeting NF-*κ*B signaling in both acute and chronic CVDs [8]. However, little is known about the exact roles of miR-181b in AS.

Human metastasis-associated lung adenocarcinoma transcript 1 (MALAT1), an 8.7 kb lncRNA on chromosome 11q13, has been demonstrated to be overexpressed in several cancers [9]. However, the roles of MALAT1 in the pathogenesis of CVDs are still not well defined. In a previous study, high expression levels of conserved MALAT1 were reported to involve in the physiological progress of endothelial cells and were related to the CVD-associated complications [10]. These lead us to investigate the roles of MALAT1 in the pathogenesis of AS.

Thymocyte selection-associated high mobility group box (TOX), which was reported to be regulated by lncRNA [11], has been closely related to the immune cell-associated proliferative diseases, such as cancer. However, whether TOX is associated with immune cell-related inflammation and oxidative stress in the progress of AS requires further clarification.

To date, rare studies have been conducted to investigate the exact interaction between MALAT1, TOX, and miRNAs. In this study, we evaluated the crosstalk between MALAT1 and TOX through investigating whether the regulatory mechanism was associated with the miRNA network.

## 2. Materials and Methods

### 2.1. Patients

Fifty AS patients and fifty healthy subjects were recruited in this study. The diagnosis was based on a history of chest pain, coronary angiography results, and characteristic ECG changes. The baseline characteristics of the two groups were compared. The peripheral blood sample (10 ml) was collected in an EDTA-containing vacutainer tube from each individual for further analysis.

### 2.2. Cell Culture

Human umbilical vein endothelial cells (HUVECs) obtained from American Tissue Culture College were cultivated in DMEM medium containing 10% fetal bovine serum (FBS, Gibco), 100 U U/ml streptomycin, and 100 U U/ml penicillin at 37°C in a humidified incubator in 5% CO_2_-95% air.

### 2.3. Cell Transfection

A miR181b mimic (Qiagen) and a modified antagomir (GenePharm) were utilized for the cell transfection in order to induce overexpression and inhibition of miR-181b in cultured cells, respectively. Transfection was conducted using the TransMessenger transfection agent (Qiagen) according to the manufacturer's instructions. A scrambled oligonucleotide (GenePharm) was used as control.

### 2.4. ROS, TNF-*α*, and NADPH Determination

ROS production in tissues and cultured cells was detected according to the previous description [12]. Initially, cells were homogenized in reaction buffer. Protein concentration was measured using the BCA method. Proteins were incubated with 20 *μ*M DCF-DA at 37°C for 3 h. The fluorescence was measured by a spectrofluorometer at an excitation of 488 nm and an emission of 525 nm, respectively. TNF-*α* level in the supernatants of HUVECs was determined using ELISA method [12]. The test was performed at least in triplicate. NADPH oxidase was also detected according to the previous description [12]. Lucigenin-enhanced chemiluminescence was used to evaluate the activity of NADPH oxidase in cell lysates with a multilabel counter (Victor 3 Wallac). In brief, 20 *μ*g protein, 100 *μ*mol/l NADPH, and 5 *μ*mol/l lucigenin were used for the assay. The procedures were conducted in the presence of DPI. Afterwards, the light signal was determined every 5 s. Finally, the NADPH oxidase activity was presented as counts per second (CPS).

### 2.5. Real-Time PCR

Total RNA was extracted from HUVECs using TRIzol reagent according to the manufacturer's instructions. The first cDNA strand was synthesized using approximately 2 *μ*g RNA with the TransScript kit (Takara), according to the manufacturer's instructions. Real-time PCR was performed based on the specific primers for MALAT1 (forward primer 5′-TCTGCAGGGACTACAGCAAG-3′; reverse primer 5′-TCACATT GGTGAATCCGTCT-3′) and TOX (forward primer 5′-TTCTCTGTG TCACCCCATGA-3′; reverse primer 5′-TCTGGCATCACAGAAATG GA-3′), using SYBR green. The mRNA level was normalized by GAPDH. The amplification results were calculated as 2(-*ΔΔ*Ct), according to the previous description [13].

### 2.6. Western Blotting Analysis

Lysis buffer containing protease and phosphatase inhibitors were utilized to extract the protein from tissues or cells. The protein was electrophoresized on a 10% SDS-PAGE gel, followed by transferring to a nitrocellulose membrane (Bio-Rad, CA, USA). Subsequently, the membrane was treated using 5% nonfat milk and then was incubated with MALAT1, TOX, ERK, and p38 antibodies overnight at 4°C. Afterwards, the mixture was incubated with the HRP-conjugated secondary antibodies at room temperature for 1 h. The same membrane probed with GAPDH served as a loading control.

### 2.7. Luciferase Reporter Assay

The luciferase vector (Addgene Inc.) including the 3′-UTR of MALAT1 and TOX containing the miR-181b response elements (wt-Luc-MALAT1 and wt-Luc-TOX) was used for the luciferase reporter assay. Site-directed gene mutation was utilized to construct a mutation in the miR-181b response elements of 3′-UTR of MALAT1 and TOX (mu-Luc-MALAT1 and mu-Luc-TOX). Subsequently, the wild and mutant 3′-UTR were cloned to the firefly luciferase-expressing vector. For the luciferase assay, HUVECs were seeded in 48-cell plates and then transfected using 200 ng plasmid DNA including wild or mutant MALAT1 and TOX, respectively.

### 2.8. Statistical Analysis

SPSS 18.0 software was used for the data analysis. The data were shown as mean ± standard deviation (SD). One-way ANOVA and parametric *t*-test were used for the intergroup comparisons. *P* < 0.05 was considered to be significant difference.

## 3. Results

### 3.1. Basic Parameters and Characteristics of Subjects in Different Groups

As shown in Table 1, total cholesterol (TC) and low-density lipoprotein (LDL-c) of patients in the AS group were higher than those of controls. Other parameters and characteristics were similar between two groups.

### 3.2. Relationship among MALAT1, miR-181b, and TOX

**Figure 1:**
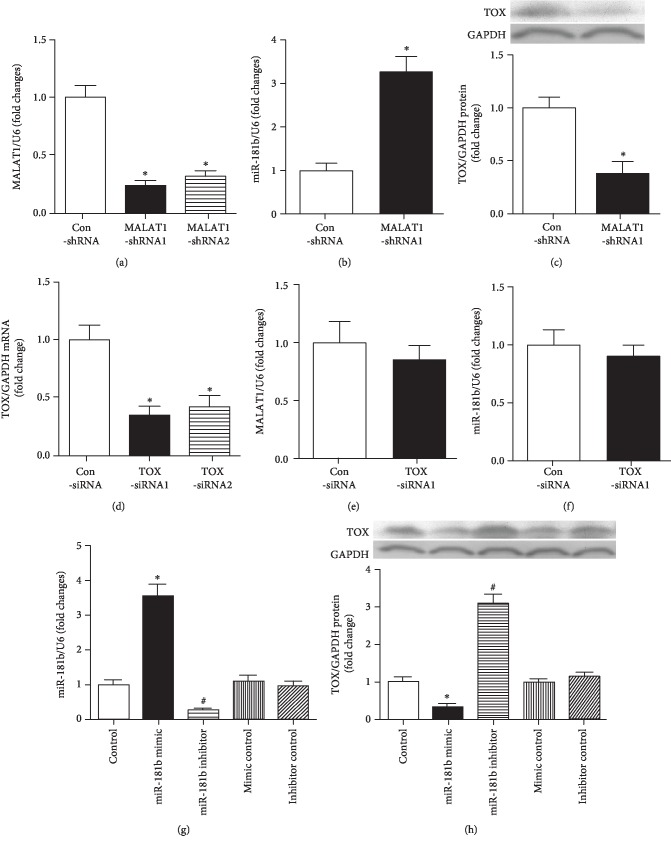
Interactions among MALAT1, TOX, and miR-181b. (a) Inhibitory effects of MALAT1-shRNAs on the MALAT1 mRNA expression as determined using RT-PCR. ^∗^*P* < 0.05 versus the control group. (b, c) HUVECs were transfected using MALAT1-shRNA1 for 24 h, followed by determining the expression of miR181b and TOX using RT-PCR and Western blot analysis, respectively. ^∗^*P* < 0.05 versus the control group. (d) RT-PCR showed TOX mRNA was downregulated after TOX siRNA. ^∗^*P* < 0.05 versus the control group. (e, f) Expressions of MALAT1 and miR181b were measured following 24 h of TOX siRNA treatment. (g, h) Alternation of miR-181b and TOX protein levels in cultured HUVECs about 24 h after various transfection treatments. ^∗^*P* < 0.05 versus the control group; ^#^*P* < 0.05 versus the control group.

Expression of MALAT1 was downregulated significantly after treating with MALAT1-shRNA1 and MALAT1-shRNA2, respectively (*P* < 0.05, Figure 1(a)). In cases of MALAT1 downregulation, the expression of miR-181b showed significant upregulation (*P* < 0.05, Figure 1(b)). Besides, after downregulation of MALAT1, TOX protein expression also showed significant decrease (*P* < 0.05, Figure 1(c)). TOX siRNA1 and TOX siRNA2 transfection could significantly downregulate the expression of TOX, especially the TOX siRNA1 (*P* < 0.05, Figure 1(d)). Then, we determined the expression of MALAT1 and miR-181b in cases of TOX siRNA1, which indicated that there were no significant changes in their expression (*P* > 0.05, Figures 1(e) and 1(f)). On the contrary, expression of TOX showed significant decrease in the presence of miR-181b mimics (Figures 1(g) and 1(h)). This implied that there might be a potential association among MALAT1, miR-181b, and TOX.

### 3.3. Expression of MALAT1 and miR-181b in AS Patients and oxLDL-Treated Cells

**Figure 2:**
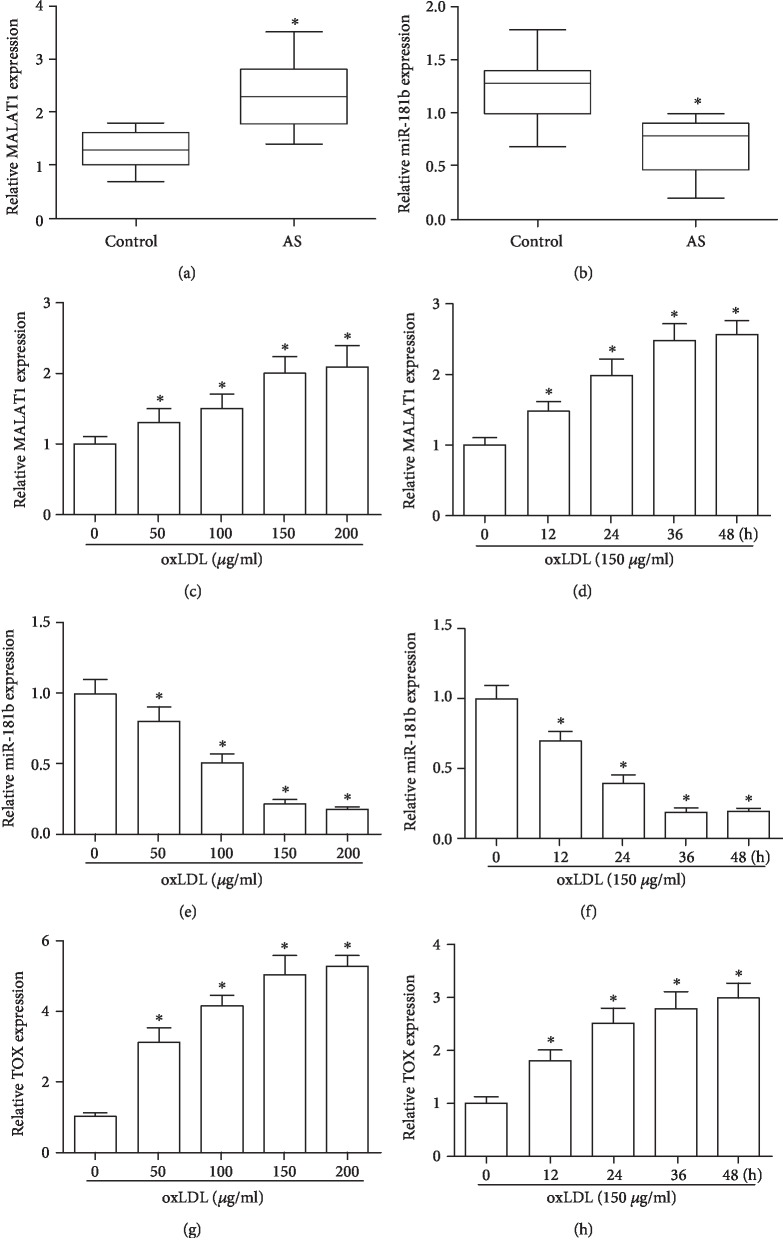
MALAT1 and miR-181b expression in AS patients and oxLDL-treated HUVECs. (a, b) Levels of circulating lncRNA MALAT1 and miR-181b in healthy volunteers and AS patients measured by RT-PCR. ^∗^*P* < 0.05 versus the control group. (c–h) Relative expression of MALAT1, miR-181b, and TOX about 24 h after treating with various concentrations of oxLDL (0, 50, 100, 150, and 200 *μ*g/ml) or treating with oxLDL (150 *μ*g/ml) for different times. ^∗^*P* < 0.05 versus the control group.

In the blood samples of AS cases, MALAT1 level showed significant increase compared with the normal individuals (*P* < 0.05, Figure 2(a)). Meanwhile, relative miR-181b expression in AS cases showed significant decrease compared with that of the normal individuals (*P* < 0.05, Figure 2(b)). Upon treating with oxLDL with different doses and times, the MALAT1 was significantly upregulated in a dose- and time-dependent manner (*P* < 0.05, Figures 2(c) and 2(d)). In contrast, the expression of miR-181b was significant downregulated in cases of oxLDL in a dose- and time-dependent manner, respectively (*P* < 0.05, Figures 2(e) and 2(f)). After treating with oxLDL with different doses and times, expression of TOX showed significant increase compared with control also in a dose- and time-dependent manner (*P* < 0.05, Figures 2(g) and 2(h)).

### 3.4. Direct Binding between miR-181b and MALAT1

**Figure 3:**
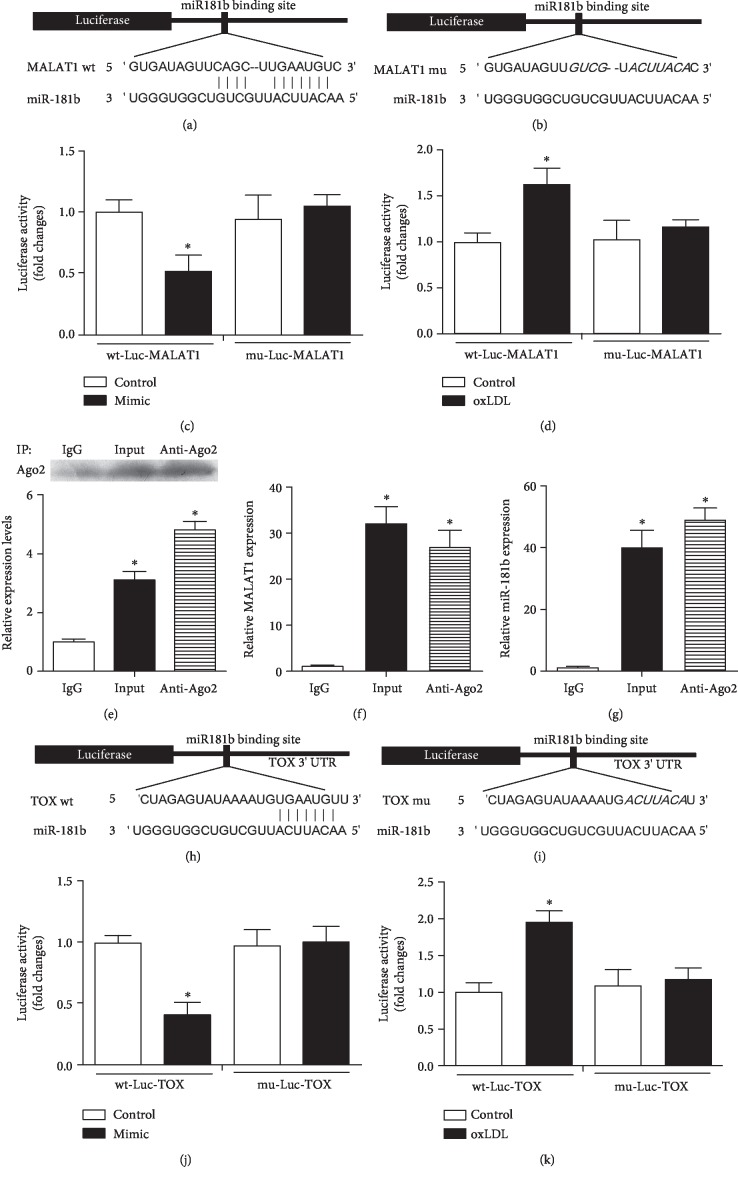
Potential binding sites of miR-181b for MALAT1 and TOX. (a, b) The wild-type and mutated miR-181b binding sites in the MALAT1 3′-UTR. (c–g) The miR-181b mimics and the luciferase constructs were cotransfected into cultured HUVECs. Cellular lysates from cultured cells were used for RIP with an Ago2 antibody. The Ago2 protein level was detected by Western blot analysis. The mRNA expression of MALAT1 and miR-181b in the immunoprecipitate was measured by RT-PCR. ^∗^*P* < 0.05 versus the control group (c, d). ^∗^*P* < 0.05 compared with IgG (e–g). (h, i) The wild-type and mutated miR-181b binding sites in the 3′-UTR of TOX. (j, k) Luciferase activity of the wild-type and mutant Luc TOX groups. ^∗^*P* < 0.05 versus the control group.

Luciferase assay indicated that miR-181b mimics induced decrease of luciferase activity of MALAT1. However, a reduced effect was found for the MALAT1 mutant (*P* < 0.05, Figure 3(a)). In the MALAT1 mutants, there were no statistical differences between the miR-181b group and control (*P* > 0.05, Figures 3(a) and 3(c)). In wild-type MALAT1, oxLDL induced significant increase of luciferase activity, while such effect was reduced after mutation in certain sites of MALAT1 (Figures 3(b) and 3(d)). In this section, we used Ago2 antibody to precipitate the Ago2 protein from cultured cells (Figure 3(e)). The mRNA expression of both MALAT1 and miR-181b was significantly enriched in the immunoprecipitates (Figures 3(c) and 3(d)). On this basis, we confirmed that there was a direct binding between MALAT1 and miR-181b.

### 3.5. Direct Binding between miR-181b and TOX

To identify the potential binding sites of miR-181b on TOX, luciferase test was performed, which indicated miR-181b mimics induced decrease of luciferase activity of TOX. However, a reduced effect was found for the mutant (*P* < 0.05, Figure 3(e)). In TOX mutants, there were no statistical differences between the miR-181b group and control (*P* > 0.05, Figures 3(f) and 3(g)). In wild type, oxLDL induced significant increase of luciferase activity, while such effect was reduced after mutation in certain sites of TOX (Figures 3(h)-3(k)).

### 3.6. Determination of TNF-*α* Expression, ROS Production, and NADPH Oxidase Activity in oxLDL-Treated Cells

**Figure 4:**
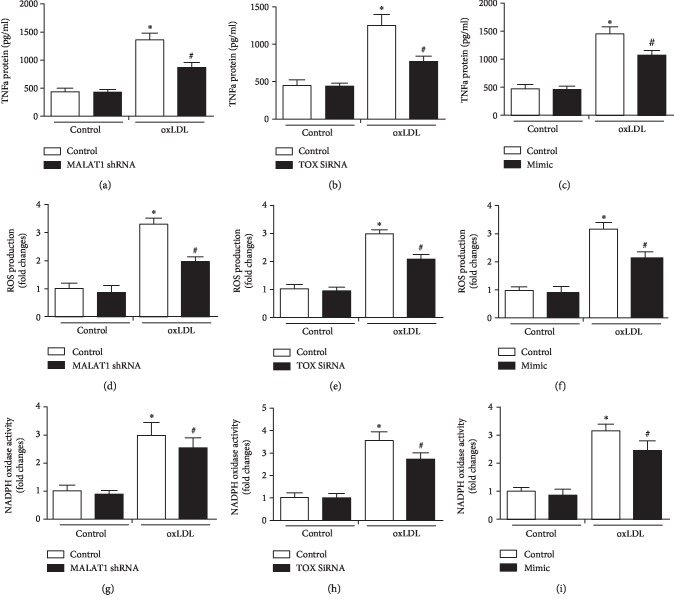
Modulation of MALAT1, TOX, and miR-181b regulated the expression of TNF-*α*, ROS production, and NADPH oxidase activity. (a–c) TNF-*α* protein expression was determined after treating with MALAT1 shRNA, TOX siRNA, and miR-181b mimic, together with ROS production (d–f) and NADPH oxidase activity (g–i). ^∗^*P* < 0.05 versus the control group; ^#^*P* < 0.05 versus the oxLDL control group.

**Figure 5:**
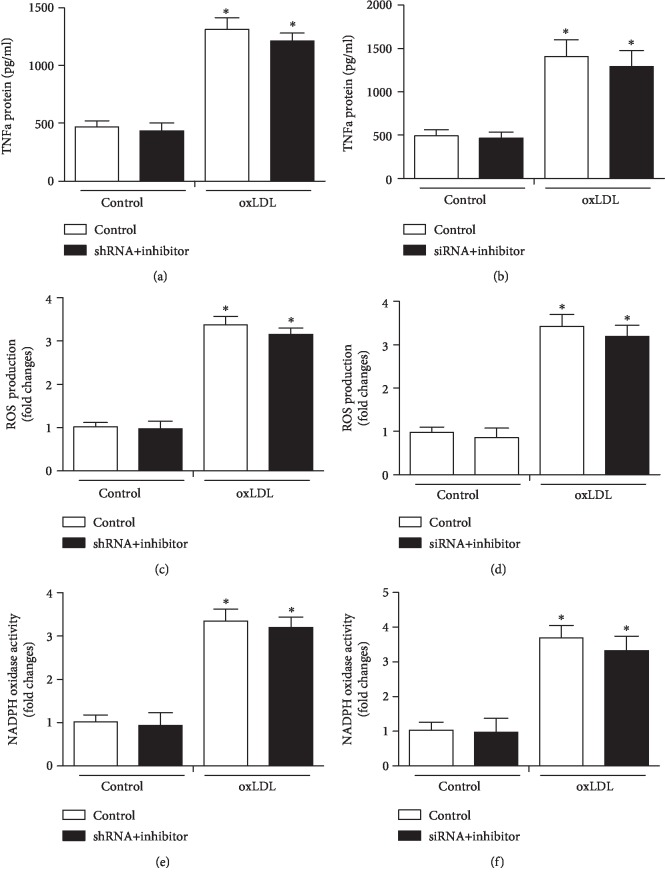
MiR-181b inhibitor in combination with MALAT1 shRNA and TOX siRNA regulated the expression of TNF-*α*, ROS production, and NADPH oxidase activity. (a–f) TNF-*α* protein expression was determined after treating with MALAT1 shRNA+miR-181b inhibitor and TOX siRNA+miR-181b inhibitor, together with ROS production and NADPH oxidase activity. ^∗^*P* < 0.05 versus the control group.

Compared with control, significant inhibition was noticed in the TNF-*α* expression, ROS production, and NADPH oxidase activity in MALAT1 shRNA, TOX siRNA, and miR-181b mimic groups in the presence of oxLDL (*P* < 0.05, Figure 4). In addition, miR-181b inhibitor usage reversed the significantly downregulating effects of MALAT1 shRNA and TOX siRNA on TNF-*α* expression, ROS production, and NADPH oxidase activity in the presence of oxLDL (*P* < 0.05, Figure 5).

### 3.7. Roles of MALAT1/miR181b/TOX in the ERK Signaling Pathway

**Figure 6:**
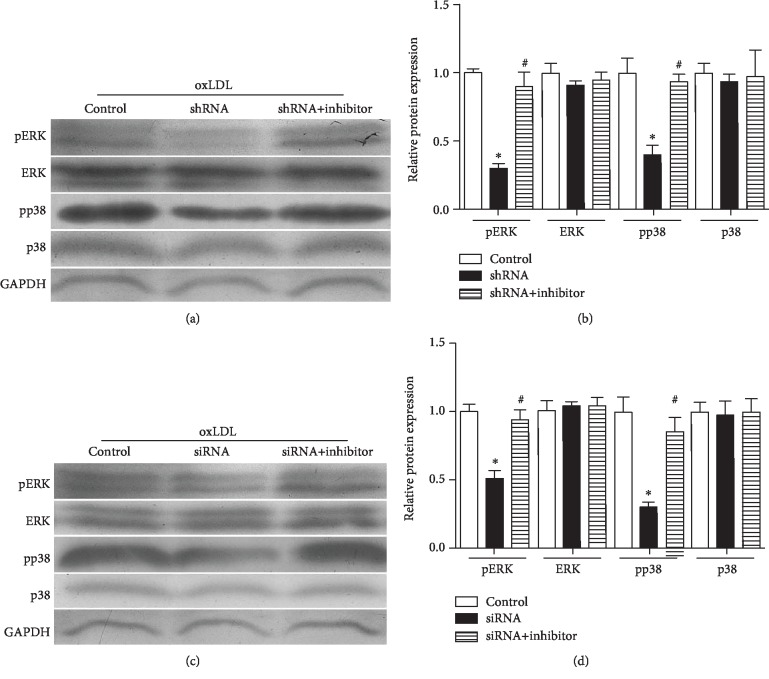
The MALAT1/miR-181b/TOX pathway is associated with the MAPK pathway. (a, b) Expression of pERK and pp38 after treating with MALAT1 shRNA and shRNA+inhibitor. ^∗^*P* < 0.05 versus the control group. ^#^*P* < 0.05 versus the shRNA group. (c, d) Expression of pERK and pp38 after treating with TOX siRNA and siRNA+inhibitor. ^∗^*P* < 0.05 versus the control group; ^#^*P* < 0.05 versus the siRNA group.

In this section, we determined the roles of MALAT1/miR181b/TOX in the MAPK signaling pathway. MALAT1 shRNA could attenuate the expression of pERK and pp38 compared with the control group (*P* < 0.05, Figures 6(a) and 6(b)). However, such phenomenon was offset in the MALAT1 shRNA+miR181b inhibitor group (*P* < 0.05, Figures 6(a) and 6(b)). TOX siRNA could significantly downregulate expression of pERK and pp38 compared with the control group (*P* < 0.05, Figures 6(c) and 6(d)). The decreased expression of pERK and pp38 in response to MALAT1 silencing could also be offset by the TOX siRNA+miR181b inhibitor (*P* < 0.05, Figures 6(c) and 6(d)).

## 4. Discussion

OxLDL and several inflammatory cytokines have been reported to be related with pathogenesis of AS [14]. According to the previous description, oxLDL deposition in endothelial barrier is also closely involved in AS [15, 16]. These reports lead us to investigate the potential targets of oxLDL, especially the downstream components of oxLDL in the development of AS, together with identification of potential lncRNAs involving in that signaling pathway.

LncRNAs have been reported to involve in regulating expression of several genes. To our best knowledge, most of the lncRNAs-related studies have been focusing on the roles of cancer progression. However, little is known about their roles in the cardiovascular diseases. In a previous study, lincRNA-p21 played an important role in the pathogenesis and progression of coronary heart disease [17]. Moreover, lncRNA APF was crucial for the regulation of autophagic cell death and myocardial infarction by targeting miR-188-3p [18]. Furthermore, it is important to investigate the crosstalk between lncRNAs and miRNAs, which may deepen our understanding on the mechanisms and pathogenesis of CVDs.

LncRNA MALAT1 is highly expressed in endothelial cells, which is closely implicated in several biological pathways. As an upstream component, MALAT1 is reported to associate with the AS. Several miRNAs including miR-101, miR-217, and miR-9 were correlated with MALAT1 [19, 20]. However, the crosstalk between MALAT1, miRNAs, and downstream targets with respect to oxLDL-induced endothelial dysfunction is still not well defined. As previously described, miRNA-181b was closely related to AS, vascular inflammation, and oxidative stress. For instance, systemic delivery of microRNA-181b inhibited vascular inflammation and AS in apolipoprotein E-deficient mice [21]. In addition, TOX plays an important role in the signaling pathways leading to CD4+ or CD8+ single-positive functionally distinct major T cell populations [22, 23], which was closely related to AS. In this study, we determined the roles of the MALAT1/miR-181b/TOX signaling pathway in the setting of AS in cases of oxLDL-induced endothelial dysfunction. In the presence of oxLDL, the expression of MALAT1 and TOX in endothelial cells was significantly upregulated presenting in a dose-dependent manner. In contrast, the expression of miR-181b was significantly downregulated in cases of oxLDL. Furthermore, it has been found that downregulation of MALAT1 could increase miR-181b expression and facilitate miR-181b-mediated TOX inhibition. These implied that there might be a potential link among MALAT1, miR-181b, and TOX. To confirm this, luciferase activity assay was performed to prove that direct bindings were existed between MALAT1 and miR-181b as well as between miR-181b and TOX. In our research, we investigated the link between MALAT1/miR-181b/TOX and inflammatory factors in the pathogenesis of CVD, by investigating TNF-*α* expression, ROS production, and NADPH oxidase activity. On this basis, we confirmed that regulation of MALAT1, miR-181b, and TOX expressions could modulate the expression of inflammatory factors that may contribute to the pathogenesis of CVD.

The MAPK signaling pathway is reported to play a physiological role in neuronal survival and endothelial cell response to atherosclerosis [24, 25]. A close link should be attached to the ERK and p38 signaling pathways in the proliferation of vascular endothelial cells and smooth muscle cells in the pathogenesis of AS. To our best knowledge, inhibition of pERK and pp38 overexpression contributed to the control of AS development. Our data showed the phosphorylation of ERK and p38 in endothelial cells showed significant decrease when exposed to MALAT1 shRNA and TOX siRNA in the presence of oxLDL. These effects were reversed by miR-181b inhibitor. On this basis, modulation of MALAT1/miR-181b/TOX may trigger downregulation of the MAPK signal pathway, which may attenuate the development of AS.

## 5. Conclusion

We presented the interactions among MALAT1, TOX, and miR-181b in oxLDL-induced endothelial dysfunction. Suppression of MALAT1 may attenuate inflammation in oxLDL-incubated endothelial cells by upregulating of miR-181b and inhibiting the expression of TOX, which is closely related to the inhibition of the MAPK signaling pathway that attenuated the pathogenesis of AS accordingly.

## Figures and Tables

**Table 1:** Basic parameters and characteristics of subjects in different groups.

	Control	AS	*P* value
*N*	50	50	ns
Sex (M/F)	31/19	27/23	ns
Age (year)	63.8 ± 7.3	65.4 ± 6.4	ns
BMI (kg/m^2^)	28.8 ± 3.0	27.3 ± 3.2	ns
TC (mmol/l)	4.14 ± 0.39	6.75 ± 0.42^∗^	<0.01
HDL-c (mmol/l)	1.45 ± 0.32	1.48 ± 0.47	ns
TG (mmol/l)	1.51 ± 0.45	1.67 ± 0.72	ns
LDL-c (mmol/l)	2.15 ± 0.37	4.46 ± 0.51^∗^	<0.01
Hypertension	24	29	ns
Diabetes	0	0	ns
History of IHD	6	8	ns
Smokers	10	10	ns

Data are expressed as mean ± SD values. AS: atherosclerosis; BMI: body mass index; TC: total cholesterol; HDL-c: high-density lipoprotein; TG: triglycerides; LDL-c: low-density lipoprotein; IHD: ischemic heart disease.

## Data Availability

The data used to support the findings of this study are available from the corresponding authors upon request.
